# Supplementary material for: A deep learning system accurately classifies primary and metastatic cancers using passenger mutation patterns
Source: Nat Commun. 2020 Feb 5;11:728. doi: 10.1038/s41467-019-13825-8 (PMC7002586; doi:10.1038/s41467-019-13825-8)
Supplement: Supplementary file 3 — Description of Additional Supplementary Files [file 41467_2019_13825_MOESM3_ESM.pdf]

Description of Additional Supplementary Files

File Name: Supplementary Data 1
Description: PCAWG Tumor Samples used for Training and Testing

File Name: Supplementary Data 2
Description: Predictive Accuracy of Random Forest Models Trained on Single Feature Categories

File Name: Supplementary Data 3
Description: Predictive accuracy of DNN trained on SNV type and distribution, mean of 10 trained models

File Name: Supplementary Data 4
Description: Distribution and source of tumour types contained within the validation data sets

File Name: Supplementary Data 5
Description: DNN classifier predictions for 62 Cancers of Uncertain Primary

File Name: Supplementary Data 6
Description: Hyperparameters selected by Bayesian optimization and their test set accuracy for classifiers trained
