# Supplementary material for: A deep learning system accurately classifies primary and metastatic cancers using passenger mutation patterns
Source: Nat Commun. 2020 Feb 5;11:728. doi: 10.1038/s41467-019-13825-8 (PMC7002586; doi:10.1038/s41467-019-13825-8)
Supplement: Supplementary file 10 — Reporting Summary [file 41467_2019_13825_MOESM10_ESM.pdf]

nature research

Corresponding author(s): Dr. Lincoln Stein NCOMMS-17-31183B

Last updated by author(s): Oct 18, 2019

Reporting Summary

Nature Research wishes to improve the reproducibility of the work that we publish. This form provides structure for consistency and transparency in reporting. For further information on Nature Research policies, seeAuthors & Referees and theEditorial Policy Checklist .

Statistics

For all statistical analyses, confirm that the following items are present in the figure legend, table legend, main text, or Methods section.

n/a	Confirmed	
☐	☒	The exact sample size (*n*) for each experimental group/condition, given as a discrete number and unit of measurement
☐	☒	A statement on whether measurements were taken from distinct samples or whether the same sample was measured repeatedly
☐	☒	The statistical test(s) used AND whether they are one- or two-sided<br>*Only common tests should be described solely by name; describe more complex techniques in the Methods section.*
☐	☒	A description of all covariates tested
☐	☒	A description of any assumptions or corrections, such as tests of normality and adjustment for multiple comparisons
☐	☒	A full description of the statistical parameters including central tendency (e.g. means) or other basic estimates (e.g. regression coefficient) AND variation (e.g. standard deviation) or associated estimates of uncertainty (e.g. confidence intervals)
☐	☒	For null hypothesis testing, the test statistic (e.g. *F*, *t*, *r*) with confidence intervals, effect sizes, degrees of freedom and *P* value noted<br>*Give P values as exact values whenever suitable.*
☐	☒	For Bayesian analysis, information on the choice of priors and Markov chain Monte Carlo settings
☐	☒	For hierarchical and complex designs, identification of the appropriate level for tests and full reporting of outcomes
☒	☐	Estimates of effect sizes (e.g. Cohen's *d*, Pearson's *r*), indicating how they were calculated

Our web collection on statistics for biologists contains articles on many of the points above.

Software and code

Policy information about availability of computer code

Data collection	No software was used
Data analysis	The code developed for training and testing the classifier, along with documentation and trained models for the 24 tumour types are available from GitHub at https://github.com/ICGC-TCGA-PanCancer/TumorType-WGS.git . The core computational pipelines used by the PCAWG Consortium for alignment, quality control and variant calling are available to the public at https://dockstore.org/search?search=pcawg under the GNU General Public License v3.0, which allows for reuse and distribution.The code is distributed under the Apache Version 2.0 Open Source license (https://www.apache.org/licenses/LICENSE-2.0).

For manuscripts utilizing custom algorithms or software that are central to the research but not yet described in published literature, software must be made available to editors/reviewers. We strongly encourage code deposition in a community repository (e.g. GitHub). See the Nature Research guidelines for submitting code & software for further information.

Data

Policy information about availability of data

All manuscripts must include a data availability statement. This statement should provide the following information, where applicable:

- Accession codes, unique identifiers, or web links for publicly available datasets
- A list of figures that have associated raw data
- A description of any restrictions on data availability

Somatic and germline variant calls, mutational signatures, subclonal reconstructions, transcript abundance, splice calls and other core data generated by the ICGC/TCGA Pan-cancer Analysis of Whole Genomes Consortium is described here7 and available for download at https://dcc.icgc.org/releases/PCAWG. Additional information on accessing the data, including raw read files, can be found at https://docs.icgc.org/pcawg/data/. In accordance with the data access policies of the ICGC and TCGA projects, most molecular, clinical and specimen data are in an open tier which does not require access approval. To access potentially identification information, such as germline alleles and underlying sequencing data, researchers will need to apply to the TCGA Data Access Committee (DAC) via dbGaP (https://dbgap.ncbi.nlm.nih.gov/aa/wga.cgi?page=login) for access to the TCGA portion of the dataset, and to the ICGC Data Access Compliance Office (DACO; http://

icgc.org/daco) for the ICGC portion. In addition, to access somatic single nucleotide variants derived from TCGA donors, researchers will also need to obtain dbGaP authorisation. The independent metastatic tumour independent validation data set generated by the Hartwig Medical Foundation is described in preprint form at https://doi.org/10.1101/415133 and is available by application to https://www.hartwigmedicalfoundation.nl/en/appyling-for-data/. The remaining metastatic and primary tumour variant call sets used for independent validation have been published and their availability is described in the publications listed in Supplementary Data 4.

Field-specific reporting

Please select the one below that is the best fit for your research. If you are not sure, read the appropriate sections before making your selection.

[x] Life sciences [ ] Behavioural & social sciences [ ] Ecological, evolutionary & environmental sciences

For a reference copy of the document with all sections, see nature.com/documents/nr-reporting-summary-flat.pdf

Life sciences study design

All studies must disclose on these points even when the disclosure is negative.

Sample size	We used genetic variants data from 2778 tumor samples from 2583 donors. The validation data set covers 1436 primary and 2090 metastatic tumors
Data exclusions	The model was trained on 24 tumor types with more than 35 samples. As a result, 342 samples in rare cancer types were excluded
Replication	We used both cross validation and external validation data set to ensure the reliability of the results.
Randomization	All samples were randomly assigned into different groups when doing cross-validation.
Blinding	Tumor type assignments from external validation data set were blinded before final valuation of the model performance.

Reporting for specific materials, systems and methods

We require information from authors about some types of materials, experimental systems and methods used in many studies. Here, indicate whether each material, system or method listed is relevant to your study. If you are not sure if a list item applies to your research, read the appropriate section before selecting a response.

Materials & experimental systems

n/a	Involved in the study	
[x]	[ ]	Antibodies
[x]	[ ]	Eukaryotic cell lines
[x]	[ ]	Palaeontology
[x]	[ ]	Animals and other organisms
[ ]	[x]	Human research participants
[x]	[ ]	Clinical data

Methods

n/a	Involved in the study	
[x]	[ ]	ChIP-seq
[x]	[ ]	Flow cytometry
[x]	[ ]	MRI-based neuroimaging

Human research participants

Policy information about studies involving human research participants

Population characteristics	We use data derived from the Pan-Cancer Analysis of Whole Genomes projects. Population characteristics are fully described in The ICGC/TCGA Pan-Cancer Analysis of Whole Genomes Network. Pan-cancer analysis of whole genomes. Nature (2019).
Recruitment	We use data derived from the Pan-Cancer Analysis of Whole Genomes projects. Patient recruitment is described in The ICGC/TCGA Pan-Cancer Analysis of Whole Genomes Network. Pan-cancer analysis of whole genomes. Nature (2019).
Ethics oversight	We use data derived from the Pan-Cancer Analysis of Whole Genomes projects. Ethics oversight is described in The ICGC/TCGA Pan-Cancer Analysis of Whole Genomes Network. Pan-cancer analysis of whole genomes. Nature (2019).

Note that full information on the approval of the study protocol must also be provided in the manuscript.
